# Public Health Messaging About Dengue on Facebook in Singapore During the COVID-19 Pandemic: Content Analysis

**DOI:** 10.2196/66954

**Published:** 2025-05-22

**Authors:** Shirley S Ho, Mengxue Ou, Nova Mengxia Huang, Agnes SF Chuah, Vanessa S Ho, Sonny Rosenthal, Hye Kyung Kim

**Affiliations:** 1 Wee Kim Wee School of Communication and Information Nanyang Technological University Singapore Singapore; 2 College of Integrative Studies Singapore Management University Singapore Singapore

**Keywords:** dengue, environment and public health, social media, Singapore, crisis and emergency risk communication, media, mosquito-borne, viruses, dengue outbreaks, epidemics, dengue transmission

## Abstract

**Background:**

Dengue, a mosquito-borne disease, has been a health challenge in Singapore for decades. In 2020, during the COVID-19 pandemic, Singapore encountered a serious dengue outbreak and deployed various communication strategies to raise public awareness and mitigate dengue transmission.

**Objective:**

Drawing on the Crisis and Emergency Risk Communication (CERC) framework, this study examines how dengue-related messages communicated on Facebook (Meta) during the COVID-19 pandemic fall into the CERC themes. This study also seeks to understand how these themes differ between dengue outbreak (eg, 2020) and nonoutbreak years (eg, 2021). In addition, we explore how message themes on dengue changed across different CERC phases within the dengue outbreak year.

**Methods:**

We conducted a content analysis on 314 Facebook posts published by public health authorities in Singapore between January 1, 2020, and September 30, 2022. We conducted chi-square tests to examine the differences in message themes between the dengue outbreak and nonoutbreak years. We also conducted chi-square tests to examine how these message themes varied across 3 CERC phases during the dengue outbreak year.

**Results:**

Our findings suggest that during the dual epidemics of dengue and COVID-19, Singapore’s public health communication on dengue largely adhered to CERC principles. Dengue-related messaging, particularly regarding intelligence and requests for contributions, significantly varied between outbreak and nonoutbreak years. In addition, messages on general advisories and vigilance, as well as those on social and common responsibility, significantly differed across the CERC phases during the dengue outbreak year.

**Conclusions:**

Singapore’s public health authorities flexibly adjusted their messaging strategies on social media platforms in response to the evolving dengue situation during the COVID-19 pandemic, demonstrating the high adaptability of the government’s health communication amid the dual epidemics. However, several areas for improvement should also be noted for future public health communication to mitigate dengue transmission.

## Introduction

### Background

Dengue, a mosquito-borne virus, is prevalent in tropical regions, with Asia accounting for 70% of cases [1]. Singapore, located in Southeast Asia, is particularly vulnerable, experiencing cyclical dengue outbreaks and remaining hyperendemic for the past 3 decades [2]. Research suggests a link between dengue and COVID-19 incidence in Asia [3], raising concerns about a dual epidemic [4,5]. Singapore experienced its most significant dengue outbreak in 2020, peaking during the COVID-19 circuit breaker period when restrictions were placed on nonessential activities [4]. In response, the government implemented various communication strategies to combat dengue during the pandemic. This study examines Singapore’s public health messaging on dengue amid the dual epidemic.

Despite the prevalence of dengue in Singapore due to reasons including environmental factors and reduced immunity levels [6], the country has managed to keep massive outbreaks under control through continuous enhancements of dengue management strategies. This is evident from the low dengue seroprevalence and consistently low force of infection rate (ie, the rate at which susceptible individuals get infected) [7] which has seen a 10-fold decline since the 1960s when strategies to manage dengue were first introduced [6].

Singapore has implemented various strategies to combat dengue, including Project Wolbachia-Singapore, which releases Wolbachia-carrying male mosquitoes to reduce mosquito populations [8]. While government efforts are crucial, public involvement is essential. To engage the community, institutions like the Ministry of Health (MOH) and National Environment Agency (NEA) tend to use Facebook, Singapore’s second most popular social media platform, to disseminate health crisis–related information [9]. However, the specific strategies used remain unclear. Understanding how governments use social media for health communication can improve issue management and enhance public adherence [10,11]. Given Singapore’s success in reducing dengue [6], studying its use of Facebook during the dual epidemic can provide valuable insights for other nations developing health communication strategies.

Drawing on the Crisis and Emergency Risk Communication (CERC) framework [12], this study has 3 objectives. The CERC framework proposes that communication during a health crisis can be divided into different phases and the communication messages across phases can be categorized into several main themes and subthemes. Specifically, this study first examines how dengue-related messages communicated on Facebook during the COVID-19 pandemic fall into the CERC themes. Second, it examines how these themes differ between the dengue outbreak (ie, 2020) and nonoutbreak years (ie, 2021) during the pandemic. Third, it also explores how message themes on dengue changed across different CERC phases within the dengue outbreak year (ie, 2020). This study contributes to the CERC literature by applying the CERC framework in a unique context—an ongoing hyperendemic disease during a pandemic. Practically, this study can help inform relevant authorities in developing effective public health messaging strategies to navigate the challenges posed by concurrent epidemics.

### The CERC Framework

This study applies the CERC framework to analyze how the Singapore government communicated dengue risks during the COVID-19 pandemic. The CERC framework [12] is well suited for this purpose, as it structures crisis communication into distinct phases, allowing strategies to adapt to evolving health crises. This framework operates on the fundamental assumption that most crises follow a largely predictable and replicable trajectory—starting with potential risks, escalating into an active crisis, and progressing toward recovery and eventual reflection or evaluation [12]. By organizing communication into clearly defined phases, the CERC framework enables risk communicators to adopt a proactive rather than reactive approach to crises, ensuring that messaging strategies are tailored to each phase for more effective communication and public response. Specifically, the CERC framework outlines five stages for tailoring communication during a health crisis: (1) precrisis stage, focusing on preparation and message development; (2) initial stage, providing immediate information to minimize risk [4]; (3) maintenance stage, offering updates and clarifications [13]; (4) resolution stage, emphasizing recovery and protective behaviors [4]; and (5) evaluation stage, reviewing postcrisis communication efforts [4,12].

The CERC framework has guided content analyses in health crises like COVID-19 [13], Zika [9], and Middle East Respiratory Syndrome [14]. Malik et al [13] expanded CERC during the COVID-19 pandemic by categorizing public communication into four themes: (1) Risk and Crisis Information, (2) Self-Efficacy and Sense-Making, (3) Preparation and Uncertainty Reduction, and (4) Advisories and Alerts. These themes include subthemes like epidemic intelligence, personal preventive measures, social responsibility, clarification, and general advisories. Building on these classifications, Malik et al [13] organize Singapore’s dengue-related Facebook content into 4 main themes and 11 subthemes, as outlined in Table 1.

**Table 1:** Definitions of Crisis and Emergency Risk Communication (CERC) message themes and examples.

CERC themes and subthemes		Definitions
**Risk and crisis information**		
	Pandemic intelligence	Messages disseminating generic, basic-level information (such as statements or numbers) about the disease, to highlight or raise awareness of the disease or the current situation [13]. In the case of dengue, this could be before, during, or after the dengue outbreak.
**Self-efficacy and sense-making**		
	Personal preventive measures and mitigation	Messages that contain measures or precautions that can be taken by an individual to protect themselves from infection, or mitigation of disease-related issues [13]. This includes mental and physical health and wellness and includes measures taken after testing or contracting the disease to prevent further transmission.
	Social and common responsibility	Messages highlighting the measures or precautions that can be taken by an individual or community to prevent the spread of the disease, for the greater good of society [13].
	Inquisitive messaging	Messages that address public queries about issues related to the disease [13].
**Preparations and uncertainty reduction**		
	Clarification	Messages intended to alert about or dispel myths, fake news, or misinformation about the disease [13].
	Events, campaigns, and activities	Messages promoting communication campaigns, events or activities for awareness, relief, or treatment of the disease [13].
	Request for contributions	Messages with a call-to-action seeking financial and voluntary contributions for tackling the disease [13].
	Showing gratitude	Messages expressing thanks, approval, regards, reassurance, and paying tribute to the frontline workers (eg, doctors, nurses, cleaners, volunteers, etc) [13].
	Reassurance	Messages that calm the public and remove their fears of the disease [13].
**Advisories and alerts**		
	Risk groups	Messages regarding people who are at greater risk of contracting the disease or experiencing the negative consequences of contracting the disease. Such messaging involves all warnings or advice pertaining to risk groups, whether it is directly to them or to the people around them [13].
	General advisories and vigilance	Messages in the form of alerts, tips, or cautions to help the public and entities in responding to the disease in certain situations, such as travel and workplace [13]. These can be presented in the form of announcements, such as the implementation of or changes in rules and regulations.

### Public Messaging About Dengue

During the COVID-19 pandemic, dengue re-emerged as a significant health threat, creating a dual epidemic in countries like Singapore, Peru, and Bangladesh [4,5]. Despite evidence linking COVID-19 and dengue in Asia [15], little research has examined how health authorities communicated dengue risks during this dual epidemic. In 2020, Singapore experienced a major dengue outbreak, with a total of 35,315 dengue cases reported [16]. Yet, studies have not explored how Singaporean authorities communicated dengue risks and preventive strategies to the public during the COVID-19 pandemic, or how these messages aligned with the CERC framework. This study aims to fill that gap by analyzing how public messaging on dengue from Singapore’s health authorities, particularly on Facebook, aligns with the themes proposed by the CERC framework.

The CERC framework has been widely applied to single emergencies, including the California measles outbreak [17], the COVID-19 pandemic [18], and natural disasters such as hurricanes [19]. Research on these cases has shown that health authorities’ communication strategies typically evolve across the phases outlined in the CERC framework, from the initial phase to resolution. Since these emergencies (eg, COVID-19 or hurricanes) have a clear and definitive endpoint, prior studies have primarily focused on examining CERC communication strategies throughout the full crisis progression, from the initial phase through to resolution.

Beyond acute emergencies, researchers have also applied the CERC framework to hyperendemic diseases, such as HIV outbreaks in Africa [20] and tuberculosis outbreaks [21]. Unlike single-crisis events, these diseases recur cyclically and lack a clear resolution, presenting unique challenges for risk communication. Previous studies have primarily focused on how CERC-based crisis communication influences behavior change [20] and its effects on public outrage during crises [21], rather than systematically comparing risk communication strategies across different CERC phases. To the best of our knowledge, existing research has yet to examine the alignment between health communication strategies and the communication principles outlined in the CERC model across different phases of hyperendemic health crises like dengue. Understanding these communication strategies would provide critical insights for improving risk messaging during hyperendemic outbreaks and contribute to refining the CERC model to better address the complexities of such recurring public health challenges.

In tropical regions like Singapore, dengue is hyperendemic, characterized by continuous transmission with intermittent significant outbreaks [6]. While dengue cases decline during nonoutbreak periods, the disease persists year-round, lacking a clear resolution or endpoint. Consequently, risk communication for hyperendemic diseases extends beyond the initial outbreak, continuing through the Maintenance to Evaluation phases [6]. Given the recurring nature of such diseases, communication strategies primarily vary across the precrisis, initial, and postcrisis phases, with the postcrisis phase incorporating elements of the maintenance, resolution, and evaluation phases outlined in the CERC framework [9]. This study, therefore, examines Singapore’s public health communication strategies during these 3 critical phases—precrisis, initial, and postcrisis—and analyzes how dengue-related messaging was conveyed across these phases during outbreaks.

In addition, the application of the CERC model to analyzing communication strategies during a dual epidemic, such as the concurrent outbreaks of COVID-19 [5], is lacking. Given dengue’s recurring nature and the unique challenges posed by dual epidemics [22], public health preparedness is crucial for early detection and timely response in such situations [2]. While intensified risk communication is essential during outbreaks, understanding communication strategies during nonoutbreak periods is equally important for establishing continuous, preventive approaches tailored to hyperendemic diseases like dengue [2,23]. These strategies include developing preemptive plans to enhance outbreak preparedness and facilitating swift, adaptive transitions when outbreaks occur [2,23].

By analyzing Singapore’s public health messaging on dengue across both outbreak and nonoutbreak periods, this study seeks to uncover how CERC strategies can be adapted to varying dengue scenarios. The findings from this study will provide valuable insights into optimizing health communication frameworks for managing both persistent and emergent public health threats, ultimately enhancing long-term disease control and crisis preparedness [2,23].

Taken together, we propose the following research questions (RQ):

- RQ1: How does Singapore’s public messaging about dengue align with the communication themes proposed by the CERC framework?
- RQ2: How do the CERC-themed messages in Singapore’s public health communication about dengue vary across dengue outbreak (ie, 2020) and nonoutbreak (ie, 2021) years?
- RQ3: How do the messages in Singapore’s public health communication about dengue vary across different CERC phases (precrisis, initial, and postcrisis) during the dengue outbreak years (ie, 2020)?

## Methods

### Overview

To achieve our objectives, we used a deductive approach in our analysis. We conducted a quantitative content analysis of Facebook messages posted by different Singapore government institutions that were involved in public health communication on dengue. These institutions include Gov.sg, MOH, Ministry of Sustainability and the Environment, NEA, and Health Promotion Board. This study focused on messages posted on Facebook, a social media platform commonly used by Singapore government institutions to communicate with the general public.

### Ethical Considerations

Before collecting data, we received ethical approval from the institutional review board (IRB) at Nanyang Technological University (IRB-2022-725) in exempt category 4. Our study involved analyzing secondary data from publicly available social media accounts, which met the exemption criteria specified under the exempt category 4. Obtaining IRB approval ensured that this study followed ethical guidelines, safeguarding the privacy and rights of individuals whose data were being analyzed.

### Data Collection and Sampling

Using the keywords “Dengue,” “dengue,” “Mozzie,” “mozzie,” “mosquito,” “breed,” “wolbachia,” “Wolbachia,” “aedes,” “BLOCK,” “B-L-O-C-K,” “SAW,” “S-A-W,” “#mozziewipeout,” “#Mozziewipeout,” “repellent,” “insecticide,” “vase,” “pails,” “pot,” and “soil,” we crawled Facebook posts of the 5 Singapore governmental institutions. We used the Python Web Crawler to crawl the Facebook posts, starting from January 1, 2020. The data collection concluded on September 30, 2022, as we needed to halt data gathering and proceed to the next phase. A total of 560 Facebook posts were collated during this period. We cleaned the data manually by removing Facebook posts that were unrelated to public health communication about dengue and the duplicates, leaving out 378 Facebook posts. Facebook posts that were excluded are posts that solely focus on the call-out to subscribe for updates, mentions of dengue as a time frame where other activities or programs are the main topic, not dengue-focused, speeches by public figures, and press releases. The data were then randomly sampled with a confidence level of 99% and a 3% margin of error, resulting in a sample of 314 Facebook posts for final analysis.

### Units of Analysis

Each Facebook post, including all text and visual elements, and everything visible on the web pages, were coded as one unit of analysis, respectively.

### Developing the Codebook

Prior to data coding, we created a codebook (see Codebook for communication strategies from the government in Multimedia Appendix 1) based on the CERC message themes adapted from existing literature [13]. The message themes included are (1) pandemic intelligence, (2) personal preventive measures and mitigation, (3) social and common responsibility, (4) inquisitive messaging, (5) clarification, (6) events, campaigns, and activities, (7) request for contributions, (8) showing gratitude, (9) reassurance, (10) risk groups, and (11) general advisories and vigilance. The definitions and examples of each code were included in the full codebook in Multimedia Appendix 1.

### Intercoder Reliability

We recruited 3 coders to code the data. The research team trained the coders before the actual coding. During the training sessions, coders were given the same units of data to code. After coding the data, they discussed their coding process to ensure a common understanding of the codebook. Once consensus was achieved, the coders received 10% of the data to code, which we used to estimate the intercoder reliability. This process was repeated twice until we achieved an average Krippendorff α value of 0.72, which is an acceptable intercoder reliability that is greater than 0.70 [24]. Subsequently, the data were split equally and coded by the coders.

## Results

Our analysis (Table 2) showed that most of the messages about dengue posted on Facebook were communicated by NEA (288/314, 91.7%), followed by the Ministry of Sustainability and the Environment (15/314, 4.8%), Gov.sg (8/314, 2.6%), Health Promotion Board (2/314, 0.6%), and MOH (1/314, 0.3%).

For RQ1, our results (Table 3) showed that most of the message themes communicated by the Singapore government were aligned with the communicated themes proposed by the CERC framework, except for inquisitive messaging (n=0). Events, campaigns, and activities were the most frequently communicated message theme (n=267), followed by personal preventive measures and mitigation (n=261), pandemic intelligence (n=231), general advisories and vigilance (n=59), social and common responsibility (n=50), and risk groups (n=16). Message themes that were less frequently communicated were clarification (n=8), showing gratitude (n=5), reassurance (n=4), and request for contributions (n=2).

**Table 2:** Distribution of Facebook posts by Singapore government institutions (N=314).

Institutions	Facebook posts, n (%)				
	2020	2021	2022	Total	
Gov.sg	1 (0.6)	2 (2.7)	5 (6.1)	8 (2.6)	
Ministry of Health	0 (0.0)	0 (0.0)	1 (1.2)	1 (0.3)	
Ministry of Sustainability and Environment	11 (6.9)	1 (1.4)	3 (3.7)	15 (4.8)	
National Environmental Agency	147 (92.5)	69 (94.5)	72 (87.8)	288 (91.7)	
Health Promotion Board	0 (0.0)	1 (1.4)	1 (1.2)	2 (0.6)	
Total	159 (100)	73 (100)	82 (100)	314 (100)	

**Table 3:** Distribution of Facebook posts based on Crisis and Emergency Risk Communication themes and Singapore government institutions (N=314).

CERC^a^ Message themes			Gov.sg		MOH^b^		MSE^c^		NEA^d^		HPB^e^		Total
**Risk and crisis information, n**													
	Pandemic intelligence	5		1		14		210		1		231	
**Self-efficacy and sense-making, n**													
	Personal preventive measures and mitigation	5		1		13		240		2		261	
	Social and common responsibility	1		0		2		46		1		50	
	Inquisitive messaging	0		0		0		0		0		0	
**Preparations and uncertainty reduction, n**													
	Clarification	0		0		1		7		0		8	
	Events, campaigns, and activities	5		0		14		246		2		267	
	Request for contributions	0		0		0		2		0		2	
	Showing gratitude	0		0		0		5		0		5	
	Reassurance	0		0		1		3		0		4	
**Advisories and alerts, n**													
	Risk groups	0		0		0		16		0		16	
	General advisories and vigilance	2		1		2		54		0		59	

^a^CERC: Crisis and Emergency Risk Communication.

^b^MOH: Ministry of Health.

^c^MSE: Ministry of Sustainability and the Environment.

^d^NEA, National Environment Agency.

^e^HPB: Health Promotion Board.

To answer RQ2, we conducted chi-square tests to examine the differences in message themes between dengue outbreak (ie, 2020) and nonoutbreak years (ie, 2021). We limited our comparison to 2020 and 2021, excluding 2022, as we only had 9 months of data for that year, making it less reliable for comparison.

The results of chi-square tests revealed that the number of messages on general advisories and vigilance was significantly different between 2020 (35/159, 22%) and 2021 (4/73, 5.5%; *χ*^2^_1_=9.8; *P*=.002). General advisories and vigilance messages were more frequently posted on Facebook during the dengue outbreak year. The number of messages on request for contributions also significantly varied between 2020 (0/159, 0%) and 2021 (2/73, 2.7%; *χ*^2^_1_=4.4; *P*=.04; Table 4). Furthermore, the differences in the number of messages on personal preventive measures and mitigation between 2020 and 2021 were almost significant (*χ*^2^_1_=3.7; *P*=.05), with the number of messages related to this theme being 138/159 (86.8%) in 2020. This was twice the number of messages (56/73, 76.7%) focusing on this theme in 2021.

For RQ3, we conducted chi-square tests to examine how CERC message themes vary across three CERC phases—precrisis, initial, and postcrisis—in 2020 (ie, the dengue outbreak year). As shown in Table 5, message themes on pandemic intelligence (*χ*^2^_2_=6.1; *P*=.047) and social and common responsibility (*χ*^2^_2_=6.1; *P*=.047) change across the CERC phases. Specifically, messages on pandemic intelligence were frequently posted on Facebook during the initial (47/69, 68.1%) and postcrisis phases (45/52, 86.5%), while messages on social and common responsibility were often posted during the initial phase (17/69, 24.6%).

**Table 4:** Comparison of Crisis and Emergency Risk Communication (CERC) message themes posted between 2020 (outbreak year) and 2021 (nonoutbreak year) (N=314).

CERC Message themes		2020, n (%)			2021, n (%)			Chi-square (*df*)		*P* value
		Yes	No	Yes		No				
**Risk and crisis information**										
	Pandemic intelligence	118 (74.2)	41 (25.8)	47 (64.4)		26 (35.6)	2.4 (1)		.13	
**Self-efficacy and sense-making**										
	Personal preventive measures and mitigation	138 (86.8)	21 (13.2)	56 (76.7)		17 (23.3)	3.7 (1)		.05	
	Social and common responsibility	26 (16.4)	133 (83.6)	6 (8.2)		67 (91.8)	2.8 (1)		.10	
	Inquisitive messaging	0 (0)	159 (100)	0 (0)		73 (100)	—^a^		—	
**Preparations and uncertainty reduction**										
	Clarification	6 (3.8)	153 (96.2)	2 (2.7)		71 (97.3)	0.2 (1)		.69	
	Events, campaigns, and activities	133 (83.6)	26 (16.4)	63 (86.3)		10 (13.7)	0.3 (1)		.60	
	Request for contributions	0 (0)	159 (100)	2 (2.7)		71 (97.3)	4.4 (1)		.04	
	Showing gratitude	1 (0.6)	158 (99.4)	3 (4.1)		70 (95.9)	3.6 (1)		.06	
	Reassurance	1 (0.6)	158 (99.4)	3 (4.1)		70 (95.9)	3.6 (1)		.06	
**Advisories and alerts**										
	Risk groups	11 (6.9)	148 (93.1)	1 (1.4)		72 (98.6)	3.1 (1)		.08	
	General advisories and vigilance	35 (22.0)	124 (78.0)	4 (5.5)		69 (94.5)	9.8 (1)		<.001	

^a^Not available.

**Table 5:** Message themes across different Crisis and Emergency Risk Communication (CERC) phases in 2020 (N=314).

CERC Message themes		Precrisis, n (%)			Initial, n (%)			Postcrisis, n (%)			Chi-square (*df*)		*P* value
		Yes	No	Yes		No	Yes		No				
**Risk and crisis information**													
	Pandemic intelligence	26 (68.4)	12 (31.6)	47 (68.1)		22 (31.9)	45 (86.5)		7 (13.5)	6.1 (2)		<.05	
**Self-efficacy and sense-making**													
	Personal preventive measures and mitigation	35 (92.1)	3 (7.9)	56 (81.2)		13 (18.8)	47 (90.4)		5 (9.6)	3.3 (2)		.18	
	Social and common responsibility	4 (10.5)	34 (89.5)	17 (24.6)		52 (75.4)	5 (9.6)		47 (90.4)	6.1 (2)		<.05	
	Inquisitive messaging	0 (0)	38 (100)	0 (0)		69 (100)	0 (0)		52 (100)	—^a^		—	
**Preparations and uncertainty reduction**													
	Clarification	0 (0)	38 (100)	4 (5.8)		65 (94.2)	2 (3.8)		50 (96.2)	2.3 (2)		.32	
	Events, campaigns, and activities	34 (89.5)	4 (10.5)	55 (79.7)		14 (20.3)	44 (84.6)		8 (15.4)	1.8 (2)		.42	
	Request for contributions	0 (0)	38 (100)	0 (0)		69 (100)	0 (0)		52 (100)	—		—	
	Showing gratitude	0 (0)	38 (100)	1 (1.4)		68 (98.6)	0 (0)		52 (100)	1.3 (2)		.52	
	Reassurance	0 (0)	38 (100)	1 (1.4)		68 (98.6)	0 (0)		52 (100)	1.3 (2)		.52	
**Advisories and alerts**													
	Risk groups	4 (10.5)	34 (89.5)	3 (4.3)		66 (95.7)	4 (7.7)		48 (92.3)	1.5 (2)		.47	
	General advisories and vigilance	8 (21.1)	30 (78.9)	16 (23.2)		53 (76.8)	11 (21.2)		41 (78.8)	0.1 (2)		.95	

^a^Not available.

## Discussion

### Principal Findings

Drawing on the CERC model, this study analyzed how Singapore’s public health authorities communicated dengue risks to the public on Facebook. Specifically, we examined how public health communication regarding dengue aligns with the message themes outlined in the CERC model. We explored how the CERC strategies used by the Singapore public health authorities vary between outbreak and nonoutbreak years, as well as the differences in the CERC strategies used across various phases within the dengue outbreak year. The findings revealed that during the dual epidemic of dengue and COVID-19, Singapore’s public health communication about dengue adhered to CERC principles. These communication strategies differed between dengue outbreak and nonoutbreak years, with a greater emphasis on requests for contributions, as well as prevention and vigilance messages, during the outbreak year as compared to the nonoutbreak year. During the dengue outbreak year, messages related to dengue cases and collective efforts were more prominent in the initial phase as compared to other phases (ie, precrisis and postcrisis).

Public health communication on dengue during the COVID-19 pandemic closely followed the message themes outlined in the CERC model, with a greater emphasis on pandemic intelligence, personal preventive measures and mitigation, and disease-related events and campaigns. These findings were consistent with previous research on the Singaporean public health communication on COVID-19 [18]. This shows that public health authorities in Singapore have put in much effort to raise public awareness about the severity of dengue and the prevention measures that the public should adopt. However, there was an absence of inquisitive messaging on Facebook, which could have been used to address the public’s inquiries about dengue-related issues. The communication of vigilant information by the public health authorities, such as general advisories and vigilance and pandemic intelligence, may contribute to increased uncertainty among the public [18,25]. A useful strategy to reduce the public’s uncertainty about crises and prevent confusion is to enhance messaging on inquisitive, especially during the initial phase. Therefore, given the lack of inquisitive messaging on dengue, our results suggest that Singapore health authorities should consider addressing the public’s questions about dengue on social media during times of uncertainty [13,25]. However, as this study only examined Singapore’s dengue-related public health messaging on Facebook, it remains unclear whether the public health authorities incorporated any inquisitive messaging in its dengue-related risk communication on other media platforms. Future research should investigate health authorities’ efforts in addressing public queries by scrutinizing risk communication messages across different platforms.

We found that dengue-related message themes varied between the dengue outbreak and nonoutbreak years. Notably, a higher proportion of alert-related messages, such as “general advisories and vigilance”, were communicated more during the dengue outbreak year as compared to the nonoutbreak year. This finding highlights the health authorities’ adaptive communication strategies in implementing alerts and regulations in response to the severe dengue outbreak. The emphasis on general advisories and vigilance aligns with the principles recommended by the World Health Organization for global vector control, such as risk-based prevention and intervention through data analytics [6]. In line with the World Health Organization principles, relevant health authorities in Singapore promptly alert stakeholders and initiate national dengue campaigns upon detecting outbreak signals, ensuring collective preparedness for outbreaks [4]. These alerting messages have also been commonly posted on social media by public health authorities in response to the outbreaks of other infectious diseases, such as COVID-19 and Ebola [4,25]. These messages can help increase public awareness about disease outbreaks and prompt immediate action, thereby helping to prevent a surge in dengue cases.

Our results also showed that the number of preventive messages conveyed by Singapore’s public health authorities on social media was significantly higher during the outbreak year (2020) as compared to the nonoutbreak year (2021), indicating a potential relaxation of dengue communication campaigns during nonoutbreak periods. However, the recurrent nature of dengue hyperendemicity requires public vigilance and preparedness, even during nonoutbreak years [26]. It would be beneficial for health authorities to also deliver dengue preventive messages during nonoutbreak periods. Maintaining consistent risk communication across outbreak and nonoutbreak years would help sustain public vigilance and better equip individuals for the potential future dengue outbreaks.

Furthermore, our study revealed variations in the CERC message themes communicated across different phases during the dengue outbreak year. The findings align with the principles of the CERC framework, suggesting that health authorities should emphasize different themes at various stages of a crisis [12]. For example, public health messaging on “pandemic intelligence” was disseminated more during the initial and postcrisis phases compared to the precrisis phase in 2020. Reports indicated a significant rise in dengue cases during the initial stage, and although the situation was brought under control in the postcrisis phase, the issue remained a key focus [16]. Therefore, our results demonstrated that the public health authorities in Singapore transparently reported the reality of dengue transmission during the initial outbreak to raise public awareness. This adherence to transparency in dengue-related communication echoes Singapore’s past risk communication strategies on Facebook during the Zika outbreak, as noted by Lwin et al [9], where authorities addressed the public’s need for information by sharing pandemic intelligence during the initial phase.

Messages about social and common responsibilities were also communicated more frequently during the initial stage, reflecting the health authorities’ efforts to emphasize the importance of community-level measures in disease prevention. The CERC model suggests that public health authorities should promote self-efficacy and encourage personal response actions [12]. One effective communication strategy is to emphasize shared responsibility among the public and stakeholders through social media [27]. By highlighting social responsibilities, individuals are more likely to actively comply with preventive measures [9,28]. This is particularly relevant in collectivist cultures, such as those in Asian societies, where social norms and community well-being are strongly emphasized [29]. Therefore, our results highlighted Singapore’s proactive adoption of CERC principles in responding to various stages of dengue outbreaks, while also considering social contexts in crisis communication.

We also found that the CERC theme—preparations and uncertainty reduction—did not differ significantly between outbreak and nonoutbreak years or across different phases. This suggests that Singapore’s public health authorities consistently engaged in risk communication on social media to prepare the public for potential dengue outbreaks, regardless of the phase or outbreak status. This finding aligns with the hyperendemic nature of dengue in Singapore, where cyclical outbreaks remain a persistent threat. Hyperendemic diseases necessitate continuous and preventive communication strategies to maintain public awareness and preparedness while enabling adaptive transitions during outbreaks [23]. The findings of this study highlight how Singapore’s public health authorities have adopted the CERC principles by consistently implementing preemptive communication plans. These efforts are critical for fostering sustained public preparedness and are essential for effectively managing hyperendemic diseases like dengue.

### Implications and Limitations

This study contributes to the literature on crisis communication during infectious disease outbreaks in 3 notable aspects. First, it adds to the body of work on CERC by being one of the first to analyze and evaluate public health communication in Singapore, a country renowned for its effective health management during both the COVID-19 pandemic and dengue outbreaks. In particular, we examined how Singapore’s health authorities applied the communication themes suggested by the CERC framework during both outbreak and nonoutbreak periods of a hyperendemic. Hence, this study provides a nuanced understanding of crisis communication principles across different epidemiological scenarios, thereby expanding the application of CERC model in the context of a hyperendemic disease. Second, this study contributes to the extant literature on public health messaging about infectious diseases by examining the communication strategies used by health authorities to address dengue within the unique context of dual epidemics.

Third, by identifying risk communication strategies within the context of a hyperendemic disease, this study highlights the need to refine the CERC framework to address the persistent and cyclical nature of hyperendemic diseases. Specifically, the absence of clear resolution and evaluation phases calls for reconsidering how to integrate continuous assessment into the framework. This adaptation could enhance the relevance of the framework for hyperendemic diseases. Our findings provide a foundation for future research to refine the CERC framework by emphasizing the importance of analyzing and comparing health authorities’ risk communication strategies across different phases of hyperendemic health crises. This includes considering both the phases outlined in the CERC model and the actual trends in disease progression. Furthermore, future research could focus on developing a modified version of the CERC framework that incorporates effective communication principles tailored to the unique characteristics of hyperendemic diseases. For example, this refined framework could highlight the importance of continuous preparation, uncertainty reduction, and long-term prevention efforts across various phases while allowing for the flexible adjustment of messaging strategies in response to disease outbreaks.

This study offers practical insights for health authorities and policymakers on how to manage a hyperendemic disease (eg, dengue), especially when faced with another epidemic (eg, COVID-19). Understanding how CERC themes were used in dengue-related communication helps identify the strengths and areas for improvement in Singapore’s risk communication strategies. For instance, our findings indicate a lack of inquisitive messaging on social media. Addressing the public questions about dengue during the initial stages of dengue outbreaks could reduce uncertainty in the future. In addition, establishing health emergency preparedness is crucial given the recurring nature of a hyperendemic (ie, dengue) [30]. During nonoutbreak periods, a proactive communication strategy focused on constant preventive messaging on social media can keep the public vigilant and prepared for potential health outbreaks [26]. Conversely, during outbreak years, policymakers could use reactive crisis response strategies, such as disseminating dengue-related advisories. As a result, health authorities and policymakers are strongly recommended to adapt these multifaceted communication strategies to different hyperendemic contexts.

While this study presented the CERC strategies used in Singapore’s public health communication about dengue during COVID-19, several limitations should be acknowledged. First, this study focused solely on dengue-related messages posted on Facebook. Dengue-related messages posted on other social media platforms, such as X and Instagram, as well as online and offline news reports and printed communication materials such as banners and brochures (which lack publication dates), were excluded. As a result, our findings may not provide a comprehensive representation of Singapore’s health messaging on dengue. The sole focus on Facebook messages may partly explain the limited representation of health authorities like the MOH in our data. Given that the MOH and other health authorities may also communicate about dengue on other platforms, such as their official websites, our data may underrepresent these institutions. Future research should consider incorporating data from a wider range of platforms and health authorities.

Second, it is important to note that this study encompassed only a portion of the Facebook messages posted in 2022, as data collection concluded on September 30, 2022. Hence, we only analyzed messages posted before that date, which may affect the interpretation of Singapore’s dengue-related communication for the full year in 2022. In addition, for statistical comparison of communication strategies between outbreak and nonoutbreak years, we only used data from 2020 and 2021, excluding the 2022 data since it only covers 9 months. To gain a more comprehensive understanding of the communication strategies adopted by health authorities and their variation across different years, future research could include Facebook posts spanning a longer time frame.

Third, this study did not evaluate the effectiveness of communication strategies by analyzing public engagement or responses to different message themes. Given that public engagement can provide valuable insights into which messages are most effective and how the public perceives government communication, future research should incorporate sentiment analysis or engagement metrics (eg, number of shares, comments, and reactions) to assess the public response to different message themes.

### Conclusions

Drawing on the CERC model, this study examined Singapore’s public health messaging about dengue during the COVID-19 pandemic. Overall, Singapore’s public health messaging about dengue was well aligned with those suggested by the CERC model. Compared to the nonoutbreak year of dengue, public health messaging during the outbreak year emphasized raising public awareness by prioritizing the dissemination of advisories and personal prevention messages related to dengue. Furthermore, public health messaging also actively provided updates about dengue cases to fulfill the public’s information needs and to encourage collective action for dengue prevention, particularly during the initial stage of the outbreak. By analyzing Singapore’s public health communication efforts about dengue, this study could provide suggestions for health authorities to effectively communicate in the hyperendemic context.

## Appendix

Multimedia Appendix 1 Codebook for communication strategies from public health authorities in Singapore.

## Abbreviations

CERC: Crisis and Emergency Risk Communication
IRB: institutional review board
MOH: Ministry of Health
NEA: National Environment Agency
RQ: research question
