# Supplementary material for: Public Health Messaging About Dengue on Facebook in Singapore During the COVID-19 Pandemic: Content Analysis
Source: JMIR Form Res. 2025 May 22;9:e66954. doi: 10.2196/66954 (PMC12141963; doi:10.2196/66954)
Supplement: Multimedia Appendix 1 [file formative_v9i1e66954_app1.docx]

**Codebook for Communication Strategies from Health Authorities in Singapore**

# ***Instructions for Coders***

1. Read through the codebook carefully.
2. Analyse each unit of analysis as a whole, with text and visual elements such as illustrations, photos, videos, etc (if applicable). Do not analyse the elements separately.
3. Code according to the requirements of each item detailed in the codebook.

# **Coding Scheme**

# ***Coder ID*** Indicate the coder ID assigned to you.

# ***Unit ID*** Indicate the unit ID as reflected on the excel sheet (data).

# ***Institution***

Identify the government institution that is hosting the unit of analysis. For example, code = “Ministry of Health” if the Ministry of Health shared content from Gov.sg.

1. **Code = “Gov.sg”** if the unit of analysis is posted or published by the **Gov.sg**
2. **Code = “Ministry of Health”** if the unit of analysis is posted or published by the **Ministry of Health**
3. **Code = “Ministry of Sustainability and Environment”** if the unit of analysis is posted or published by the **Ministry of Sustainability and Environment**
4. **Code = “National Environmental Agency”** if the unit of analysis is posted or published by the **National Environmental Agency**
5. **Code = “Health Promotion Board”** if the unit of analysis is posted or published by the **Health Promotion Board**

Theoretical Concepts

Identify and indicate the constructs present in the units of analysis based on the theoretical concepts below.

4. Crisis and Emergency Risk Communication (CERC)

1. Refer to the bold and/or underlined words and/or phrases in the examples under each corresponding column for clarity on how to classify a unit of analysis to the code.
2. **Code = “Absent”** if the sub-codes under the theme are **absent** in the unit of analysis
3. **Code = “Present”** if the sub-codes under the theme are **present** in the unit of analysis

Does this unit of analysis, i.e., Facebook post/ webpage/ poster/ brochure, illustrate….?

1. Risk and crisis information
   1. Pandemic intelligence
2. Self-efficacy and Sense-making
   1. Personal preventive measures and mitigation
   2. Social and/or common responsibility
   3. Inquisitive messaging
3. Preparations and uncertainty reduction
   1. Clarification
   2. Events, campaigns and activities
   3. Request for contributions
   4. Showing gratitude
   5. Reassurance
4. Advisories and alerts
   1. Risk groups
   2. General advisories and vigilance

| **Themes** | | | |
| --- | --- | --- | --- |
| **Risk and crisis information -** General public understanding of associated risks  Note: this theme does not cover messages that concerns misinformation or counterarguments to misinformation. | | | |
| **Codes** | | **Definition** | **Examples** |
| 1. Pandemic intelligence | | Messages that contain generic, basic-level information (such as statements or numbers) about the disease, to highlight or raise awareness of the disease or the current situation. | **“**“Dengue gave me high on-and-off fever, pain in my eyes, headaches, body aches, and skin rashes. The most uncomfortable part were the body aches, which seemed to worsen every time I tried to move. "  **About 60% of Aedes mosquito breeding detected are from homes.** Practice the B-L-O-C-K steps to remove all stagnant water and keep the mozzies away!  📣We’d like to hear your story if you or your loved ones have caught dengue before! Simply comment in the section below, or on your own social media page with the tag #BLOCKDengue, and you may be featured on our page.” [Example from data] |
| **Themes** | | | |
| **Self-efficacy and Sense-making** – Feedback processes to make sense of the situation and changes in behaviors to reduce the likelihood of harm | | | |
| **Codes** | | **Definition** | **Examples** |
| 3. Personal preventive measures and mitigation | | Messages that contain measures or precautions that can be taken by an individual to protect themselves from infection, or mitigation of disease-related issues. This includes mental and physical health and wellness, and includes measures taken after testing and/or contracting the disease to prevent further transmission. | “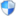Protect yourself and your loved ones with these 6 important steps! 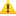  1. Wear long sleeves and long pants  2. Apply mosquito repellent regularly  3. Turn over pails when not in use  4. Spray insecticide in dark corners  5. Close windows when leaving home  6. Flip over flowerpot plates  For more, go to go.gov.sg/fight-dengue”  [Example from data] |
| 4. Social and/or common responsibility | | Messages that contain measures or precautions that can be taken by an individual or community to prevent the spread of the disease, for the greater good of the society | “**Urgent stepped-up community action is needed against dengue.** With close to 5,800 dengue cases in 2020 as at mid-Apr, the number of dengue cases is more than double that in the same period last year. Unless immediate action is taken to suppress the Aedes mosquito population, the number of dengue cases is expected to exceed the 16,000 cases noted in 2019.  There is also a 50% increase in mosquito breeding found in homes over the past 3 years, compared to in the preceding 3 years. Clean stagnant water, even as small in volume as the size of a 20-cent coin, can be potential mosquito breeding habitats.  **As more of us are working from home during this circuit breaker period, pay more attention to potential mosquito breeding habitats, and do the #MozzieWipeout as part of your daily routine. If your family is experiencing more mosquito bites at home, you should also spray aerosol insecticide in dark corners, such as under the bed, behind the cupboard, and in the toilet, to kill any adult female mosquitoes.** Here’s a short video which shows where you should spray insecticides at home: www.facebook.com/watch/?v=2870377276333638  **All of us have a part to play in preventing dengue!**  More information available at http://www.nea.gov.sg/.../dengue-cases-expected-to-exceed...” [Example from data] |
| 5. Inquisitive Messaging | | Messages that address public queries about issues related to the disease. | N.A. |
| **Themes** | | | |
| **Preparations and uncertainty reduction** – Preparing the public for the possibility of an adverse event | | | |
| **Codes** | | **Definition** | **Examples** |
| 6. Clarification | | Messages intended to alert about or dispel myths, fake news, or misinformation about the disease. | “A Facebook post **claiming that drinking lemon juice can help to prevent Dengue fever** has been making the rounds. This claim has been **debunked** by the Ministry of Health and **is not true.”** [Example created] |
| 1. Communication campaigns, events and activities | | Messages promoting communication campaigns, events or activities for awareness, relief, or treatment **of the disease**. Communication campaigns refer to efforts to raise awareness and change the behavior of the audience in a designated time frame through different messages that are informational and persuasive across a variety of media platforms (Atkin and Rice, 2013). | “Wozzie mozzie cannot fight dengue alone, he needs your help!  Help him fight dengue by doing the Mozzie Wipeout BLOCK steps and protect yourself from bites with the SAW steps. We need to continue our efforts as a community, to keep our neighbourhoods and homes free from mosquitoes and dengue. Stay tuned for Episode 10 to find out how Project 𝘞𝘰𝘭𝘣𝘢𝘤𝘩𝘪𝘢 is working!  Find out more about Project 𝘞𝘰𝘭𝘣𝘢𝘤𝘩𝘪𝘢 at go.gov.sg/wolbachia  #ProjectWolbachiasg” [Example from data] |
| 1. Request for contributions | | Messages with a call-to-action seeking financial and voluntary contributions for tackling the disease. | “The fight against dengue never stops, even during Phase 2 and 3 Heightened Alert! Our dengue prevention volunteers have continued to distribute educational materials to encourage residents living in dengue clusters to do the B-L-O-C-K Mozzie Wipeout steps. All while making sure to adhere to existing safe distancing guidelines. A big "Thank You" to our dengue prevention volunteers for helping to keep our community safe from dengue during this period! **Join us as a dengue prevention volunteer** by signing up at https://www.cgs.gov.sg/volunteer today!” [Example from data] |
| 1. Showing gratitude | | Messages expressing thanks, approval, regards, reassurance, and paying tribute to the frontline workers (e.g., doctors, nurses, cleaners, volunteers, etc). | “The fight against dengue never stops, even during Phase 2 and 3 Heightened Alert🚨! Our dengue prevention volunteers have continued to distribute educational materials to encourage residents living in dengue clusters to do the B-L-O-C-K Mozzie Wipeout steps. All while making sure to adhere to existing safe distancing guidelines.  A big "Thank You" to our dengue prevention volunteers for helping to keep our community safe from dengue during this period! 👏🏼😊  Join us as a dengue prevention volunteer by signing up at https://www.cgs.gov.sg/volunteer today!  #BLOCKDengue #MozzieWipeout #SGCleanAmbassadors”  [Example from data] |
| 1. Reassurance | | Messages that calm the public and removes their fears of the disease. | “Meet Wozzie the Wolbachia bacterium! Dengue mosquito numbers are coming down in Choa Chu Kang and Bukit Batok towns, thanks to Wozzie and support from the community!  As part of Project Wolbachia, male Wolbachia-carrying Aedes mosquitoes, or Wozzie Mozzies, are released every Tuesday and Friday morning in parts of Choa Chu Kang and Bukit Batok towns. Continued releases will help reduce the dengue mosquito population in the community. Don’t worry, male Wozzie Mozzies do not bite. Watch to find out how they help fight dengue!  Remember, Project Wolbachia is not a silver bullet. Let’s do our part to fight dengue by practising the Mozzie Wipeout to keep our homes mosquito-free.  For more information, visit go.gov.sg/Wolbachia  #ProjectWolbachiasg” [Example from data] |
| **Themes** | | | |
| **Codes** | **Definition** | | **Examples** |
| **Advisories and alerts** – specific advice or warning messages regarding the disease | | | |
| 1. Risk groups | Messages regarding people with pre-existing conditions or seniors who are at greater risk of contracting the disease or experiencing the negative consequences of contracting the disease. Such messaging involves all warnings or advice pertaining to risk groups, whether it is directly to them or to the people around them. This includes messages that suggest that risk groups need to be alert to certain elements of the disease or that the people around them should be mindful and protect them. | | “It's 24/7 today! Who are the ones you'd be around 24/7 for? Being a parent is a 24/7 job and what hurts more than seeing your child in pain?  Do the #MozzieWipeout, apply mosquito repellent, wear long-sleeved clothing and trousers and spray insecticide in dark corners around your home to protect your family, especially if you live in a dengue cluster (www.nea.gov.sg/dengue-zika/dengue/dengue-clusters) or in an area with higher Aedes aegypti mosquito population (www.nea.gov.sg/dengue-zika/Aedes). [Example from data] |
| 1. General advisories and vigilance | Messages in the form of alerts, tips or cautions to help the public and entities responding to the disease in certain situations such as travel and workplace. These can be presented in the form of announcements such as implementation or changes in rules and regulations.  Keywords to look out for include: announced, advise, urge, recommend, precaution, take steps, take action, seek (e.g., cooperation to), eligible to, comply, encouraged | | “Detecting and removing mosquito breeding habitats and mosquito larvae or pupae are key to curbing dengue transmission. However, during NEA’s recent inspections, we continue to detect premises with multiple mosquito breeding habitats, and profuse mosquito breeding.  For instance, we found profuse mosquito breeding at residential premises located in the Clover Ave, Arnasalam Chetty Road/Kim Yam Road and Aljunied/Geylang Road dengue clusters. In one of these homes, there were a few hundred larvae detected in a water feature.  #NEAOfficers will continue with our inspections, especially at dengue cluster areas, to protect the community. From 15 July 2020 onwards, **enhanced penalties will be also imposed** for households, construction sites and Town Councils found to be breeding mosquitoes. You can also **take these preventive steps** to protect yourself and your family from dengue: https://youtu.be/JU_pM1_Uls8  #DenguePrevention #MozzieWipeout”  [Example from data] |
